# Applicability of a shortened interpretation model for intraoperative parathyroid hormone monitoring in patients with primary hyperparathyroidism in an endemic goiter region

**DOI:** 10.1007/s10353-018-0547-8

**Published:** 2018-07-11

**Authors:** Philipp Riss, Angelika Geroldinger, Andreas Selberherr, Lindsay Brammen, Julian Heidtmann, Christian Scheuba

**Affiliations:** 1 Section of Endocrine Surgery, Division of General Surgery Department of Surgery, Medical University of Vienna, Waehringer Guertel 18–20, 1090 Vienna, Austria; 2 Section for Clinical Biometrics, Center for Medical Statistics, Informatics, and Intelligent Systems (CeMSIIS), Medical University of Vienna, Vienna, Austria

**Keywords:** Primary hyperparathyroidism, Intraoperative parathyroid hormone monitoring, Vienna criterion, Parathyroid surgery, Surgery

## Abstract

**Background:**

In primary hyperparathyroidism (pHPT), quick intraoperative parathyroid hormone monitoring (IOPTH) is performed to predict complete excision of hyperfunctioning tissue and therefore cure. In recent years, efforts have been made to make this prediction more accurate and to shorten the duration of the test, respectively, and therefore reduce waiting and total operating time. The aim of this study was to evaluate the practicability and safety of a time-reduced criterion (decline ≥ 35% after 5 min) in a large cohort of patients.

**Methods:**

In an 11-year period, all patients operated for pHPT were analyzed. After preoperative localization studies, hyperfunctioning parathyroid tissue was removed and IOPTH monitoring was performed. Intraoperatively, a decline of ≥50% from baseline 10 min after excision of the gland predicted cure. The performance of an interpretation model, using an earlier PTH level was analyzed retrospectively (decline ≥ 35% from baseline 5 min after excision). Differences in sensitivity, specificity, positive/negative predictive value and accuracy were calculated.

**Results:**

According to the inclusion criteria, 1018 patients were analyzed. IOPTH predicted cure in 854 patients (83.9%) 10 min after gland excision with a false positive decline in 13 patients (1.5%). Applying the modified criterion (≥35% decline within 5 min), 814 patients (80%) showed an appropriate decline (false positive in 18 [2.2%]). Overall, multiple gland disease would have been missed in 7 patients. McNemar’s test showed a significantly lower sensitivity, specificity and accuracy applying the “35%” criterion.

**Conclusions:**

In an endemic goiter region, a criterion, demanding a ≥ 35% decline 5 min after excision can not be recommended for IOPTH monitoring in patients with pHPT.

## Introduction

In patients with primary hyperparathyroidism (pHPT), surgical removal of hyperfunctioning parathyroid tissue is the treatment of choice. Thus, altered calcium homeostasis can be normalized and, as recently published, also oxidative stress reduced [1]. Preoperatively localized enlarged gland by ultrasound and sestamibi scintigraphy (together with a single photon emission computed tomography with x-ray computed tomography [SPECT/CT]) allow minimally invasive (targeted/focused) procedures. However, in endemic goiter regions, concomitant thyroid surgery is necessary in 47% of the patients (unilateral or bilateral exploration due to hemi-/thyroidectomy) [2]. Nevertheless, intraoperative PTH monitoring (IOPTH monitoring) is also used to confirm complete excision of hyperfunctioning tissue in these patients, as multiple gland disease cannot be ruled out in preoperative localization studies. IOPTH also led to the term “biochemical frozen section”, as intraoperative histological proof (frozen section) seems not to be regularly necessary [3]. Different interpretation criteria for the intraoperative PTH curve are described in the literature. They mainly rely on a drop of ≥50%, either from the pre-incision value or the highest pre-excision value, respectively, within 10 min after excision of the enlarged gland. It has been shown that different interpretation criteria may have an altered performance in an endemic goiter region [4]. Reasons for that remain unclear.

Despite the excellent long-term results of parathyroid surgery (reported cure rates of >98%), efforts have been made to shorten the waiting time for IOPTH results and therefore to reduce length of surgery. Alhefdhi et al. recently published a criterion requiring a 35% drop within 5 min after gland excision, which is the first described shortened criterion for the interpretation of the intraoperative PTH decay [5]. The aim of this study was to evaluate this criterion in a large cohort of patients.

## Materials and methods

Among all 1120 consecutive patients operated on for sporadic primary hyperparathyroidism in an 11-year period in a tertiary referral center, 1018 were included. Exclusion criteria were reoperation (*n* = 31), parathyroid carcinoma (*n* = 2), hereditary disease (*n* = 21), or technical issues (missing PTH values; *n* = 48). Long-term follow-up was performed in all patients. Cure was defined as permanent normocalcemia. “Persistence” was diagnosed if calcium levels (together with inappropriately high PTH values) were measured within 6 months and as “recurrence” if measured after 6 months.

Following a standard protocol [2], open minimally invasive surgery was performed if ultrasound and/or sestamibi scan localized a single enlarged gland. Unilateral or bilateral neck exploration, respectively, was performed in patients with concomitant hemi-(thyroidectomy) or negative/discordant localization studies.

## Intraoperative PTH monitoring

The first blood sample was drawn after induction of anesthesia but before skin incision and defined as “baseline” value. Further samples were drawn after excision of the gland, 5 and 10 min afterwards. A decay ≥ 50% from baseline within 10 min after excision of the gland predicted cure.

The data were reanalyzed by applying the time-reduced criterion, requiring a decay ≥ 35% from baseline within 10 min after excision of the gland.

The study was approved by the local ethics committee (No. 1610/2015).

## Statistical analyses

Sensitivity, specificity and accuracy of the different criteria were calculated. For each of these measures, exact 95% confidence intervals (CI) were calculated. To compare sensitivity and specificity the McNemar test was used. Differences were considered significant if *p* was less than 0.05. Statistical calculations were performed using the SAS system V 9.4 (SAS Institute Inc., Cary, NC, USA).

## Results

A total of 1018 patients with biochemically proven hyperparathyroidism were included: 814 women (80%), 204 men (20%). Median age was 62 years. Preoperative laboratory findings are documented in Table 1.

**Table 1:** Preoperative biochemical analyses of 1018 patients

	Median (quartiles)	Normal range
Serum calcium	2.77 (2.66, 2.93)	2.20–2.55 mmol/l
PTH	131.8 (96.9, 194.4)	15–65 pg/ml
Creatinine	0.89 (0.79, 1.01)	<1.2 mg/dl
24 h urinary calcium	7.17 (4.44, 9.83)	2.5–7.5 mmol/24 h

*PTH* parathyroid hormone

A total of 619 patients (60.8%) underwent minimally invasive open exploration or unilateral exploration; in 325 (31.9%) bilateral neck exploration was necessary. A conversion (unilateral to bilateral exploration due to false localization) was necessary in 71 patients (7%). An intrathoracic adenoma was removed in 3 patients (0.3%). In all, 952 patients had single gland disease (93.5%) and 63 (6.2%) multiple gland disease (double adenoma or four-gland hyperplasia). In 3 patients (0.3%), despite bilateral extended exploration including transcervical thymectomy, central neck dissection and dissection of the carotic sheath, respectively, no adenoma could be found (negative exploration). Altogether, long-term follow up showed persisting disease in 10 patients (1%).

### Standard criterion

The criterion, requiring a decay ≥ 50% from baseline within 10 min after excision of a hypersecreting parathyroid gland, was reached by 854 patients (83.90%). Thirteen of 854 patients (1.52%) had a false positive (FP) decline (decline despite having further hypersecreting tissue in situ), 841 patients had a correct decline (true positive [TP]). In 164 patients (16.11%) an inappropriate decline indicated further hypersecreting tissue. This was correct in 60 patients (true negative [TN]) and incorrect in 104 (false negative [FN]).

### “35%” criterion

The adapted criterion requires a ≥ 35% decline from baseline within 5 min after excision. According to this criterion, 814 patients (79.96%) had an appropriate decline. Eighteen of 814 patients (2.21%) had an FP and 796 (97.79%) had a correct decline (TP).

A total of 204 patients (20.04%) did not have an appropriate decline indicating further hypersecreting tissue in situ. This was correct in 55 patients (26.96%; TN) and incorrect in 149 (73.04%; FN). The sensitivity, specificity and accuracy of the “35% criterion” were 0.84, 0.75, and 0.84, respectively (Table 2).

**Table 2:** Sensitivity, specificity, PPV, NPV, and accuracy (proportion and 95% confidence interval) of the two different criteria

	Decline ≥ 50% (within 10 min)	Decline ≥ 35% (within 5 min)
Sensitivity	0.89 (0.87–0.91)	0.84 (0.82–0.87)
Specificity	0.82 (0.71–0.90)	0.75 (0.64–0.85)
PPV	0.98 (0.97–0.99)	0.98 (0.97–0.99)
NPV	0.37 (0.32–0.42)	0.27 (0.23–0.31)
Accuracy	0.89 (0.86–0.90)	0.84 (0.81–0.86)

*PPV* positive predictive value, *NPV* negative predictive value

### Standard vs. “35%” criterion

Applying the “35% criterion”, 814 patients (79.96%) showed a positive decline allowing to discontinue surgery. However, 7 patients with MGD would have been missed. McNemar’s test showed a significant difference in the sensitivity (*p* < 0.0001) as well as in the specificity (*p* = 0.0078) comparing the standard criterion with the “35%” criterion.

## Discussion

Modern parathyroid surgery is performed with great success, as cure rates of >98% are described in the literature [4, 6]. Preoperative localization studies with ultrasound and sestamibi (performed with SPECT/CT) together with IOPTH monitoring made minimally invasive, focused/targeted surgery possible. However, efforts are made to even enhance performance with new localization techniques (i.e. PET/CT)[7, 8] and improvement of IOPTH monitoring. The latter is possible by shortening the duration time of the assay and developing more efficient interpretation criteria. Since the introduction of IOPTH monitoring by Irvin et al. [9], different interpretation models have been described—each with particular advantages and drawbacks [3, 4, 10, 11]. Most criteria require a drop ≥ 50% from baseline (pre-incision value) [4] or from the highest per-excision value [9], respectively, within 10 min after excision of the gland. Stricter criteria demand a drop into the (lower) normal range showing a lower rate of missed MGD (higher specificity) [3, 12] but lead to a higher number of unnecessarily extended explorations (lower sensitivity), and vice versa. Recently, Alhefdhi et al. [5] documented that a 5 min decline of 35% can distinguish single adenoma from multiple gland disease with a high sensitivity and specificity. This would combine both a reliable criterion and a shorter waiting time for the IOPTH results. However, it has been shown previously that performance of interpretation criteria can differ in an endemic goiter region [4]. This study therefore aimed to evaluate if the good results of this criterion are reproducible in a large cohort of patients, operated in an endemic goiter region.

As standard interpretation criterion in the analyzed patients, a decay of ≥50% from baseline (pre-incision value) 10 min after excision of the gland was used in this study. A good discrimination between SGD and MGD in an endemic goiter region has been reported for this criterion [4]. Accordingly, sensitivity was 0.89 and specificity 0.82, respectively, in this study. The most important aim of IOPTH monitoring is the detection of MGD. Therefore, a high specificity is necessary in order not to miss patients with more than one hypersecreting gland. Garbutt et al. [13] also documented that using post-induction but pre-incision value as baseline lead to the highest overall accuracy compared to other baseline values. Applying the “35%” criterion, specificity was significantly lower (0.75) and seven additional patients with MGD would not have been cured. Sensitivity, which was 0.84 for this interpretation model, mainly depends on FN assay results. FN results are often a consequence of intraoperative manipulation of the gland and the subsequent PTH spike, which can lead to a prolonged PTH decline [14–16]. However, these spikes can be recognized intraoperatively (careful interpretation of the decay) and do not necessarily lead to an unnecessary more extended exploration. Therefore, the success rate for criteria with high specificity but lower sensitivity is also acceptable. It remains unclear if larger [16] or smaller adenoma [17] lead to a more extensive PTH spike. Nevertheless, Carr et al. [16] showed in an analysis of 900 patients that higher PTH spikes more likely occur in SGD, whereas in patients with MGD a less extensive PTH excretion can be observed intraoperatively.

There is still a debate if IOPTH monitoring is necessary even in patients with localized SGD and no evidence of MGD in preoperative localization studies [6, 18, 19]. Still, sestamibi and ultrasound are not good in detecting MGD and, furthermore, it was documented that without IOPTH monitoring, the number of patients with persisting disease would significantly increase [18]. This was recently confirmed by Bobanga et al. [6]. Another study, with only “selective” use of IOPTH also reported a higher rate of persisting disease (5% without vs. 0% with IOPTH) [20]. Moreover, IOPTH may be a reliable marker for predicting parathyroid carcinoma, as documented by Dobrinja et al. [21].

Nevertheless, applying both criteria used in this study, the high number of FN results documents that the perfect criterion has not been found yet and further studies in this field seem to be necessary.

## Limitations of the study

Main limitation is the drawback of a retrospective analysis. Furthermore, the applicability of the 35% criterion was analyzed theoretically, as intraoperatively decisions were made according to the results of the standard criterion.

## Conclusion

Analyzing a large cohort of patients, a criterion, demanding a ≥ 35% decline 5 min after excision cannot be recommended for IOPTH monitoring in patients with primary hyperparathyroidism.
